# Evidence for a missing source of efficient ice nuclei

**DOI:** 10.1038/srep39673

**Published:** 2017-01-03

**Authors:** Rui Du, Pengrui Du, Zedong Lu, Weishan Ren, Zongmin Liang, Saisai Qin, Ziming Li, Yaling Wang, Pingqing Fu

**Affiliations:** 1 College of Resources and Environment, University of Chinese Academy of Science, Beijing 100049, China; 2 State Key of Laboratory of Atmospheric Boundary Physics and Atmospheric Chemistry, Institute of Atmospheric Physics, Chinese Academy of Sciences, Beijing 100029, China

## Abstract

It has been known for several decades that some bioaerosols, such as ice-nucleation-active (INA) bacteria, especially *Pseudomonas syringae* strains, may play a critical potential role in the formation of clouds and precipitation. We investigated bacterial and fungal ice nuclei (IN) in rainwater samples collected from the Hulunber temperate grasslands in North China. The median freezing temperatures (*T*_50_) for three years’ worth of unprocessed rain samples were greater than −10 °C based on immersion freezing testing. The heat and filtration treatments inactivated 7–54% and 2–89%, respectively, of the IN activity at temperatures warmer than −10 °C. We also determined the composition of the microbial community. The majority of observed *Pseudomonas* strains were distantly related to the verified ice-nucleating *Pseudomonas* strains, as revealed by phylogenetic analysis. Here, we show that there are submicron INA particles <220 nm in rainwater that are not identifiable as the known species of high-INA bacteria and fungi and there may be a new potential type of efficient submicroscale or nanoscale ice nucleator in the regional rainwater samplers. Our results suggest the need for a reinterpretation of the source of high-INA material in the formation of precipitation and contribute to the search for new methods of weather modification.

Since the middle of the last century, there has been a substantial body of work showing the importance of bioaerosols as ice-nucleating particles (INPs) in mixed-phased and ice clouds, ultimately affecting the local, regional and global climate and precipitation^1^,^2^,^3^,^4^,^5^,^6^,^7^. With the increasing importance of microbiological meteorology, scientists in disparate fields have led a resurgent attempt to understand the mechanisms of biological IN in which supercooled water droplets are transformed into ice^8^. Thus far, several works have not only elucidated the mechanism through which molecular biology influences the ability of known biological IN to initiate freezing at warmer temperatures in the laboratory^9^,^10^,^11^ but also isolated ice-nucleation-active (INA) bacteria from many different environments^12^,^13^,^14^,^15^,^16^. Furthermore, researchers have reported a new species of INA fungus, *Mortierella alpina,* which initiates freezing above −6 °C^17^. However, as the atmospheric importance of INA bacteria is questionable^18^,^19^, their role in the formation of clouds and precipitation in the real atmosphere has been debated. Recently, several works have attempted to investigate the quantities of INA genes^20^, submicroscale INA bacterial fragments^16^ and even nanoscale biological IN^21^,^22^,^23^ to demonstrate that biological ice nuclei (IN), especially the best-known efficient IN species from the genera *Pseudomonas, Erwinia, Xanthomonas* and *Fusarium,* are widespread in the atmosphere. However, to date, the apparent contradiction between the low ratio of biological to inorganic IN and the importance of their function has not been settled. Moreover, thus far, the research into ice nucleation in the atmosphere is based on cloud chamber-like experiments and individual drop isolation techniques. It is difficult to reproduce atmospheric conditions in the laboratory for evaluating the importance of bioaerosols in atmospheric processes that result in precipitation. Thus, the role of biological IN in precipitation is still elusive. In particular, the amount and types of efficient biological IN as well as whether biological IN dominate the natural atmospheric highly efficient IN solely at temperatures higher than −10 °C in the natural environments.

In the current study, we applied immersion freezing tests to predict whether biological efficient IN are omnipresent in rainwater samples based on a comparison of the initial and median freezing temperature of droplets exposed to filtration and heat treatments. Furthermore, the microbial communities and phylogenetic relationship were determined using 16S RNA and 18S rRNA gene analysis to elucidate whether there are known ice-nucleating bacteria and fungi present in the samples collected from the Hulunber temperate grasslands in north China.

## Results

### Unusual median and onset freezing temperatures of rain samples

**Figure 1:**
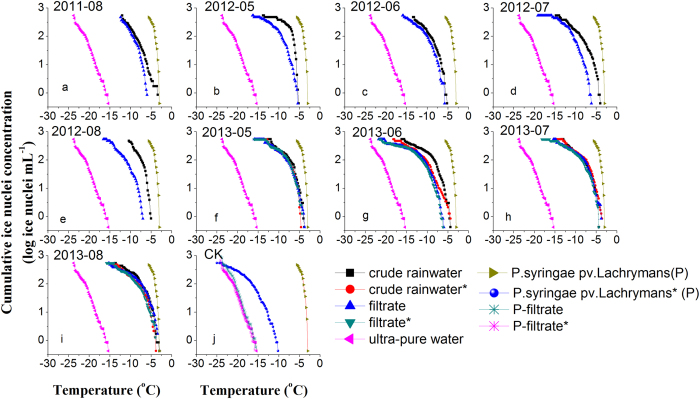
Cumulative IN spectrum of ultra-pure water, *P. syringae pv. lachrymans* suspension and rainwater samples collected from the Hulunber grassland from 2011 to 2013. Notes: *P. syringae pv. lachrymans* refers to the *P. syringae pv. lachrymans* solutions, P. syringae pv. lachrymans* refers to the *P. syringae pv. lachrymans* solutions under heat treatment, P-filtrate refers to the filtrated *P. syringae pv. lachrymans* solution, P-filtrate* refers to the filtrated *P. syringae pv. lachrymans* solution under heat treatment.

**Figure 2:**
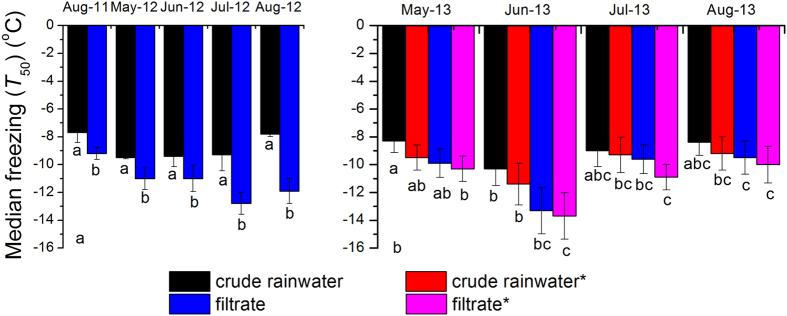
Median freezing temperature (T_50_) of rainwater samples collected from 2011 to 2013 under different treatments. Notes: * refers to crude rainwater, filtrate and particle suspension samples exposed to heat treatment. The superscripts **a**, **b** and **c** above the bars denote significantly different values (P < 0.05).

Figure 1 shows the cumulative spectra of IN per unit volume of the nine rain samples collected from August 2011 to August 2013 as well as of the ultra-pure Milli–Q water and a suspension of *P. syringae pv. Lachrymans*^24^ (*PS*) suspension (10^8^ cells mL^−1^) as controls. *PS* initiated freezing at −3.0 °C and produced 10^2.6^ IN per milliliter at −5.6 °C, at which the freezing process of all droplets ended; the ultra-pure Milli–Q water initiated freezing at −15.4 °C. Two crude rainwater samples (August 2011 and August 2013) were active at about −3.0 °C, while others induced freezing at −3.6 °C to −5.2 °C. The results of T_50_ values of the crude rainwater were −7.7 °C (August 2011), −9.5 °C (May 2012), −9.4 °C (June 2012), −9.3 °C (July 2012), and −7.8 °C (August 2012) (Fig. 2a). We assumed that these findings resulted from the widespread distribution of the known species of INA bacteria and fungi in the rainwater^13^,^14^,^24^. Filtration led to the complete elimination of the ice-nucleating bacterium *PS* (10^8^ cells mL^−1^) at temperatures above −10 °C (Fig. 1j). But, both crude rainwater droplets and filtered rainwater droplets triggered freezing above −7 °C (Table 1). As a result of the filtration treatment, the initial freezing temperature decreased by around 0.2–2.6 °C (Table 2) and T_50_ decreased around 1.5–4.1 °C for the five rain samples in 2011 and 2012 (Fig. 2a). Filtration treatment reduced 16–73% of biological and non-biological IN active at temperature ≥ −10 °C. However, the freezing temperature of the filtrate was close to −10 °C or higher. The prevailing view is that most IN active at warmer than −15 °C in clouds could be biological particles^25^ and INA of biological IN is more efficient than that of inorganic substances^26^. However, both INA bacteria and fungi as efficient INPs because they can synthesize INA proteinaceous that are heat sensitive materials^14^,^20^,^27^. And so, heat is a simple but effective tool to disrupt and denature these proteinaceous INPs. Unexpectedly, the heating treatment had little effect on the tested samplers. Compared to the crude rainwater, heat treatment led to a slight decrease (0.3‒1.2 °C) in freezing temperature (Fig. 2b) and a decrease of 0.2‒1.0 °C in the onset freezing temperature at which freezing initially occurred (Fig. 1f–i, Table 2).

It is noteworthy that almost efficient INPs both from marine^28^ and terrestrial sources^13^,^14^,^20^,^22^,^27^ are thermally labile materials that are heat denatures proteins and causing a reduction in activity by heating treatment. Whereas, there seem to be certain efficient IN in our test samplers but it is not heat sensitive.

### Presence of novel efficient INPs in rain samplers

The known biological INPs induce ice to nucleate heterogeneously at temperatures warmer than is common for heterogeneous ice nucleation. From the spectra, the crude rain samples are similar and show intermediate ice nucleation activity between the suspension of *PS* and the ultra-pure Milli–Q water. The ice nucleation activity of the *PS* suspension was several orders of magnitude higher than all rain samples over the temperature range between −3.0 and −5.0 °C (Fig. 1). In addition to the drop-freezing assay, DNA analysis by Sanger sequencing was carried out for these particles >220 nm in the 9 rainwater samples. No isolated strain culture test was carried out. A total of 908 bacterial clones and 470 fungal clones were generated across all samples. The most abundant genus bacteria was *Massilia* (32% of total Sanger sequence sets) followed by *Acinetobacter* (15%) and *Janthinobacterium* (13%), while *Pseudomonas* only accounted for 4% of the bacterial sequences and the genera *Erwinia* and *Xanthomonas* were not found in the rainwater. Moreover, *Pseudomonas*, composed of numerous species of highly IN active bacteria, was detected in four samples (July 2013, July 2012, August 2012 and August 2011), accounting for 25%, 2%, 6%, and 7% of each sample sequence, respectively.

The fungi community structures on a genus level were different in the all rainwater samples. The greatest amount of the fungus sequences were identified as *Cryptococcus* (40% of the fungal sequences in rainwater). The genera *Fusarium* and *Mortierella*, also known as efficient fungal IN, were also not found in the rain samples. These findings are not consistent with previous reports on the widespread distribution of *Pseudomonas spp*. in aerosol, cloud and fog samples^29^,^30^,^31^,^32^,^33^,^34^. However, Mortazavi *et al*.^35^ also found that none of eight types of bacterial species isolated from snow belonged to known effective ice nucleators such as *Pseudomonas* or *Erwinia* genera^35^.

**Figure 3:**
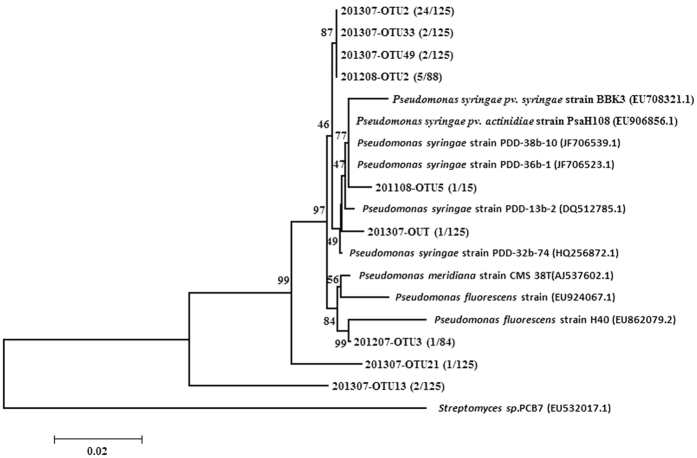
Neighbor-joining phylogenetic tree constructed on the basis of Sanger sequences with similarities to the 16S rRNA gene of known ice-nucleating bacteria. The tree was rooted on the Streptomyces sequence.

To explore the role of the biological IN in present study, a close phylogenetic relationship among the 16S rRNA gene sequences of the identified *Pseudomonas* strains and many of the verified ice-nucleating *Pseudomonas* species, *P. syringae*^36^, *P. fluorescens*^26^, *P. meridian*^26^, was confirmed (Fig. 3). The results show that the known ice-nucleating bacteria are not common in our detected Sanger sequences. Consequently, we speculate that there may be a new potential type of efficient submicroscale or nanoscale ice nucleator that does not belong to previously known highly efficient INA biological species in the samplers.

## Discussion

In order to verify and interpret above noted phenomenon, rain samples collected from May to August in 2013 were further tested for nucleation abilities. A comparison of the cumulative IN concentration spectra in four rain samples exposed to different treatments revealed a similar concentration range of IN (Fig. 1f–i). The T_50_ values of the crude rainwater were −8.3 °C (May 2013), −10.3 °C (June 2013), −9.0 °C (July 2013), and −8.4 °C (August 2013) (Fig. 2b). Compared to crude rainwater in 2013, the heat treatment reduced the IN concentration by 24–62% at −6 °C, 28–78% at −8 °C, and 9–54% at −10 °C. The T_50_ value (−9.2 °C to −11.4 °C) was no longer significant after heat treatment (P > 0.05) (Fig. 2b), and the freezing temperature of droplets mainly lay between −10 °C and −5 °C (see Supplementary Fig. S1). Our measurement results are not consistent with previous reports on the effect of heat on biological INA of environmental samples^13^,^14^,^15^,^16^,^20^,^27^,^28^,^37^. In fact, hitherto, it has been proposed that efficient biological IN is proteinaceous and easily effected reducing IN activity by heat treatment. In contrast, although the INA of pollen and inorganic substances is not heat labile at close to 100 °C^26^,^38^,^39^, they also do not belong to effective ice nuclei. Additionally, the T_50_ values of the filtrates are −9.9 °C (May 2013), −13.3 °C (June 2013), −9.6 °C (July 2013), and −9.5 °C (August 2013) (Fig. 2b). For example, heat treatment caused a decrease of 1.0 °C in the initial freezing temperature and 1.2 °C in the median freezing temperature, and only 16% of total IN 237 IN mL^−1^ active at ≥ −10 °C were sensitive to heat treatment for the May 2013 sample. Meanwhile, as a control experiment, heat treatment led to the complete elimination of ice nucleation at temperatures above −10 °C in the ice-nucleating bacteria *PS* (10^8^ cells mL^−1^) (Fig. 1j). Therefore, Hara *et al*.^37^ suggested a method of estimating biological IN concentrations in environmental samples based on their heat tolerance. The authors inferred that the obviously decreased IN concentrations above −10 °C temperature were due to the presence of biological INA substances and could classify biological IN components in environmental samples based on their heat sensitivity following different heating temperature^37^.

Compared to the heat treatment, filtration should let to the complete elimination of the INA bacteria at least intact cells. However, the May 2012 rain sample initiated freezing at the highest temperature (−5.4 °C) after filtering with 39 and 143 IN per milliliter at −8 °C and −10 °C, respectively (Fig. 1b and Table 1). Furthermore, the Aug 2012 rain sample initiated freezing at the lowest temperature (−7.0 °C) after filtering with 7 and 48 IN per milliliter at −8 °C and −10 °C, respectively (Fig. 1e and Table 1). Bacteria and other particles larger than 220 nm were removed by filtration through a polycarbonate filter. Actually, the results of Neighbor-joining phylogenetic tree (Fig. 3) indicate that only a few sequences (201207–OTU3) are closely related to *Pseudomonas fluorescens* strain H40 (accession number EU862079.2) with a 16S rRNA gene sequence identity of 97%. Sequence 201108–OTU5 falls into the branch that is closely related to the *Pseudomonas syringae pv. actinidiae* strain PsaH108 (EU906856.1) and *Pseudomonas syringae* strain PDD–38b–10 (JF706539.1). The 16S rRNA gene sequence (201108–OTU5) shows 97% identity with *Pseudomonas syringae pv. actinidiae* strain PsaH108 and 97% identity with *Pseudomonas syringae* strain PDD–38b–10. Sequence 201307–OTU39 shows 98% identity with *Pseudomonas syringae* strain PDD-13b-2 (DQ512785.1). The 16S rRNA-gene-based phylogeny show that sequences 201208–OTU2, 201307–OTU2, 201307–OTU33 and 201307–OTU49; sequence 201307–OTU21; and sequence 201307–OTU13 all belong to a new branch. Therefore, almost of the discovered famous INA bacteria stains do not have ice nucleation activity. Although the INA fungal proteins were considerately less heat-sensitive than bacterial INA, they were also inactive above −10 °C after heating at 90 °C^22^,^27^. Both the bacterial and fungal IN were inactivated above −10 °C by heat treatment at 100 °C for 10 min, while the IN of other origins were not. Thus, the reduction in IN concentration following heat treatment should represent the total pool of bacterial and fungal IN in a sample. Even though Šantl-Temkiv *et al*.^16^ demonstrated that the known INA bacteria can be abundantly present in precipitation as submicron fragments <0.2 μm, rather than as intact cells, and initiate freezing^16^. Furthermore, several results showed that INA fungal proteins would pass through 0.22-mm-pore membrane filters^22^. Given limitation of the technology detection sensitivity of Sanger sequencing technique, there may be some submicron fragments <0.2 μm of the others famous INA microorganism in the rain samplers filtrate. But, the T_50_ values of the heating filtrates are −10.3 °C (May 2013), −13.7 °C (June 2013), −10.9 °C (July 2013), and −10.0 °C (August 2013) (Fig. 2b), respectively. Also, compared to the filtrate, heat treatment decreased the onset freezing temperature by 0.2–0.8 °C, all still induced freezing above −6 °C and resulted in a 0–61% decrease in the abundance of IN between −6 °C and −10 °C (Table 2) and decreased non-significantly T_50_ by approximately 0.3–1.3 °C (P > 0.05) (Fig. 2b). On the contrary, previous studies have found that on average, 69–100% of biological efficient ice nucleation was deactivated by heat treatment at 95 °C for 10 min in precipitation collected from locations around the world^12^,^13^. Complementary, O’Sullivan *et al*.^22^ recently also suggested that the known INA fungus *F. avenaceum* may produce nano-INPs that attach to soil particles and are thus transferred to the atmosphere, can function independently of fungal cells, and are nanometer in scale and thus easily pass through a 0.22-μm-pore membrane. These particles are smaller than 200 nm and not active at temperatures above −10 °C after heating to temperature 90 °C or higher^22^. Similarly, the marine biogenic INPs such as exudates of both marine diatom *Thalassiosira pseudonana* and phytoplankton cells are also smaller than 200 nm and can’t keep activity above −10 °C due to their heat sensitivity. Thence, they are not the likely candidate for the observed efficient IN.

In summary, due to the higher freezing temperature of the filtrate, these submicron IN are not likely to be the known biological IN fragments because of their tolerance to heating at 100 °C. Besides, potassium feldspar, as the only inorganic INA particle, has comparable INA to biological particles at temperatures above −8 °C and is not sensitive to heat treatment^38^. Unfortunately, kaolinite, rich in potassium feldspar, could not likewise induce freezing at −3.4 °C and −4 °C (Tables 1 and 2). Thus, we concluded that there may be a new submicron or nanoscale efficient IN in the Hulunber grassland rainwater filtrate that is neither a known biological IN nor the inorganic IN K- feldspar.

This result indicated that missing efficient IN most likely exist in addition to biological IN. Little is known of the sources, abundance, and spectra of the IN activities of this novel submicroscale element. We used relatively simple tests such as filtration, heat treatment, and DNA analysis as well as droplet-freezing experiments for rapid screening of biogenic submicroscale-INPs in natural rainwater samples. Rangel-Alvarado *et al*.^21^ investigated the nanosized particles present in North American snow, an important form of precipitation, and characterized their physical, chemical, and biological properties using a suite of modern laboratory techniques. Their results illustrated that particles smaller than 200 nm are dominant and account for 38–71% all snow-borne particles. Such particles have relatively high freezing temperature, ranging on average from −19.6 ± 2.4 to −8.1 ± 2.6 °C with a mean freezing temperature of −17.2 ± 7.1 °C^21^. The results of chemical analysis of the nanosized fraction showed that they are mainly composed of amino acids, monomers and peptides. Additionally, the authors could not eliminate the roles of viruses and nanodust particles in the ice-nucleation processes^21^. Additionally, to date, there is no evidence that the presence of more efficient IN in the immersion mode excludes the presence of efficient proteinaceous IN produced by *P. syringe* and *F. avenaceum* with heat tolerance above 100 °C.

In the atmosphere, primary biological aerosol particles play important roles in atmospheric chemistry and physics, but their role in ice formation remains poorly understood. Microorganisms are not geographically restricted, and meteorological conditions influence biological ice nucleation. Consequently, it is important to understand the concentration and physical chemistry of bioaerosols and combine this knowledge with atmospheric modeling to evaluate their climatic importance. In our study, only *Pseudomonas* was found in rainwater samples collected during Jul 2013, Jul 2012, Aug 2012 and Aug 2011 from the Hulunber Grassland, Inner Mongolia, China. The known bacterial IN genera *Erwinia* and *Xanthomonas* and the known fungal IN genera *Fusarium* and *Mortierella* were not detected. Interestingly, the average *T*_50_ of the three years of filtered rainwater with particles smaller than 220 nm was still −10.9 ± 1.4 °C (n = 9) and the freezing temperature of droplets mainly ranged from −10 °C to −5 °C. These IN are not heat sensitive and are not the known efficient IN. This discovery is exciting because it raises the possibility of a novel INA material in the environment that has not been considered in models. This result may thus facilitate our understanding of the possible ice-nucleation mechanisms of submicroscale or nanoscale particles. These results can also expand our previous understanding of efficient IN materials in the natural environment and contribute to the field of weather modification by presenting a candidate for efficient IN.

Conservatively, we suggest that the existence of a missing biological or non-biological heat-resistant efficient IN of <220 nm in rainwater had a maximum effect on ice nucleation between −10 °C and −5 °C. Future research should focus on understanding the nature of this substance as an IN. In particular, its chemical composition, physical structure and environmental distribution should be investigated.

## Methods

### Sampling sites and preparation

Rain samples were collected at a grassland site at 49°19′N, 120°03′E, 628 m a.s.l., in the Hulunber Grassland Ecosystem Research Station of the Chinese Academy of Agricultural Sciences in Inner Mongolia, China, from 2011–2013. This region is semi-arid, and the annual mean precipitation is 400 mm (150–550 mm), with a large inter-annual variation, falling primarily between June and August. Rain samples were collected in sterilized 3-L beakers equipped with sterilized homemade stainless steel rain collectors. The collector was shaped much like a funnel, with a wide outer region twice the diameter of the beaker, and installed on top of the beaker to collect as much rainwater as possible during precipitation events. The beakers were placed in open areas at 1.5–2 m height to avoid splashing from plants and the ground surface and were rinsed once with rainwater before being set for collection. It was difficult to meet the required sample volume of >1 L in one rain event. The rain samples were therefore collected throughout one month on the dates indicated in Table 3. Each sample was immediately stored in a refrigerator at 4 °C prior to processing. All of the materials used for sampling were sterilized by autoclaving, and a sterile mask and gloves were worn during sample collection to avoid any potential contamination. The rain samples referred to in this paper are well-mixed rainwater, collected within a one-month period.

A crude rain volume of 0.5 L was filtered through two sterile nitrous cellulose filters with a pore diameter of 0.22 μm (Millipore, USA) to be used for DNA extraction and the associated microbial community composition analyses. The crude rainwater samples and filtrate were then transferred to a sterile plastic container. The well-mixed crude sample was aliquoted into two 10-mL test tubes. One tube was heated to 100 °C using a water bath for 10 min to disrupt the structure of membrane-bound proteins. The remaining tube was a control, which was subjected to an immersion freezing test. The same methods described above were also applied to the filtrate and particle suspension. The reference strains of *P. syringae pv lachrymans (PS*) were provided by the State Key Laboratory for Biology of Plant Diseases and Insect Pests in the Chinese Academy of Agricultural Sciences. Suspensions of *PS* (10^8^ cells mL^−1^) were obtained with the cultivated bacteria in sterilized ultra-pure Milli-Q water^24^. As a control, the suspensions of *PS* were also subjected to heat and filtration treatment.

### Drop-freezing assays

The immersion freezing tests were performed using a modified device based on Vali’s method^40^. Forty-seven 10-μL droplets were equally distributed on a sterile copper plate with a cooling rate of 2 °C min^−1^, with five repetitions, for a total of 235 droplets tested in each sample^24^,^41^. The initial temperature was 0 °C, which was then reduced at a rate of 2 °C min^−1^ until all droplets froze. This modified device could automatically detect the drop-frozen signals and calculate the experimental data^42^. In brief, the top of device is an enclosed cubicle that prevents dust contamination. The 49 thermo-sensitive elements (7 lines × 7 rows × 7 elements, where rows were 10 mm long and elements 10 mm wide) adhered to the temperature-controlled working plate (140 mm long × 140 mm wide × 5 mm high) using a set of thermocouples with proportional spacing in the cubicle. One thermocouple was affixed with a small high-precision Pt100 sensor for temperature measurement, and another acted as a signal reference. The plate surface was washed with acetone and then coated with hydrophobic film before the 47 droplets were placed 18 mm apart^42^,^43^. The temperature of the working plate was decreased at an approximate rate controlled by a Eurotherm 818P4 temperature controller. The latent heat released by the freezing droplets was monitored in real time by a computer and transformed into a voltage signal.

Ice-nucleation data analysis. Throughout the paper, *T*_50_ refers to the temperature at which 50% of the droplets froze. The cumulative IN concentration at each temperature was calculated according to the following equation^40^:




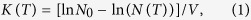




Where, *K(T*) is the cumulative concentration of IN at temperature *T, N*_*0*_ is the number of droplets tested, *N(T*) is the cumulative number of unfrozen droplets at a given temperature *T* and *V* is the volume of the drop.

### DNA extraction and PCR amplification

Microbial DNA was extracted from the samples using the Fast DNA spin kit (Bio101, Qbiogene Inc., CA, USA) according to the manufacturer’s instructions. The gene fragments from the extracted total DNA were amplified using polymerase chain reaction (PCR) as follows: 95 °C for 5 min, followed by 35 cycles at 95 °C for 1 min, 55 °C for 1 min, 72 °C for 1.5 min, and a final extension at 72 °C for 10 min using the 16S rRNA gene universal primer 27 f (5′–AGAGTTTGATCCTGGCTCAG–3′) and 1492r (5′–ACGGCTACCTTGTTACGACTT–3′), and the fungal ITS1–5.8S-ITS2 forward primer ITS1f (5′–CTTGGTCATTTAGAGGAAGTAA–3′) and reverse primer ITS4 (5′–TCCTCCGCTTATTGATAT–3′). The PCR reactions were performed in a 50-μL mixture containing 5 μL of 10× PCR buffer, 4 μL of 2.5 mM dNTPs, 2.0 μL of each primer (10 μM), 0.5 μL of BSA (10 mg mL^−1^), 1.0 μL of template DNA, 0.5 μL of rTaq (5 U μL^−1^) and 35 μL of sterile ultrapure water. The PCR products were electrophoresed on a 1% (w/v) agarose gel.

### Cloning and sequencing

The PCR product was purified using a GeneJET PCR Purification Kit (Fermentas, USA). Purified PCR products were ligated into a pGEM–T Easy vector, transformed into DH5α *Escherichia coli* cells following the manufacturer’s instructions (Takara Bio Inc., Japan) and sequenced at Majorbio Bio-Pharm Technology Co. (Shanghai, China). All sequences were checked for chimeric artifacts using the Mallard program. Clones with more than 97% sequence similarity were grouped into the same operational taxonomic unit. The representative sequences were compared to those available in the GenBank databases using the basic local alignment tool nucleotide (BLASTN) through the National Center for Biotechnology Information server. The bacterial 16S rRNA gene sequences and fungal 18S rRNA gene sequences obtained were deposited in GenBank under accession numbers KU514494 to KU515401 and KU515483 to KU515952, respectively. Phylogenetic trees were constructed with the neighbor-joining method using MEGA 4.0^44^. Bootstrap resampling analysis for 1000 replicates was performed to estimate the confidence of the tree topologies.

### Statistical analysis

Statistical analyses were carried out using SPSS (version 12.0, SPSS Inc., Chicago, USA) and Origin 8.0 (Origin Lab Corporation, USA). Differences between treatments were analyzed with an independent-samples test or one-way ANOVA depending on the sample population being investigated.

## Additional Information

**How to cite this article:** Du, R. *et al*. Evidence for a missing source of efficient ice nuclei. *Sci. Rep.* **7**, 39673; doi: 10.1038/srep39673 (2017).

**Publisher's note:** Springer Nature remains neutral with regard to jurisdictional claims in published maps and institutional affiliations.

## Supplementary Material

Supplementary Figure S1

## Figures and Tables

**Table 1:** Total IN concentration and decreased proportion of IN in crude rainwater under filtration treatment at −6 °C, −8 °C, and −10 °C.

Sample	Onset freezing temperature (°C)	Onset freezing temperature after filtration treatment (°C)	Decrease of onset freezing temperature by filtration treatment (°C)	IN mL^−1^ [total (% filtration -sensitive)]		
				Temperature (°C)		
				−6	−8	−10
2011-08	−3.2	−5.8	2.6	11 (92)	66 (43)	202 (30)
2012-05	−5.0	−5.4	0.4	26 (100)	256 (85)	385 (63)
2012-06	−5.2	−5.8	0.6	5 (55)	44 (50)	147 (31)
2012-07	−3.6	−6.2	2.6	18 (100)	71 (85)	161 (58)
2012-08	−5.0	−7.0	2.0	16 (100)	151 (95)	436 (89)
2013-05	−3.6	−3.8	0.2	32 (33)	143 (33)	237 (13)
2013-06	−4.2	−4.8	0.6	13 (97)	85 (85)	190 (66)
2013-07	−3.6	−4.4	0.8	26 (18)	109 (7)	184 (2)
2013-08	−2.8	−3.4	0.6	52 (1)	155 (18)	252 (12)
PS	−3	−15.6	12.6	326 (100)		

**Table 2:** Total IN concentration and decreased proportion of IN of solutions (crude and filtrate rainwater) under heat treatment at −6 °C, −8 °C, and −10 °C.

Sample	Onset freezing temperature (°C)	Onset freezing temperature after heat treatment (°C)	Decrease of onset freezing temperature by heat treatment (°C)	IN mL^−1^ [total (% heat-sensitive)]		
				Temperature (°C)		
				−6	−8	−10
Crude rainwater						
2013-05	−3.6	−4.6	1.0	32 (26)	143 (28)	237 (16)
2013-06	−4.2	−4.4	0.2	13 (62)	85 (78)	190 (54)
2013-07	−3.6	−4.4	0.8	26 (24)	109 (22)	184 (9)
2013-08	−2.8	−3.8	1.0	52 (41)	155 (39)	252 (28)
Filtrate						
2013-05	−3.8	−4.4	0.6	21 (17)	96 (17)	206 (13)
2013-06	−4.8	−5.6	0.8	0 (0)	13 (39)	65 (18)
2013-07	−4.4	−4.6	0.2	21 (19)	101 (32)	180 (7)
2013-08	−3.4	−4.0	0.6	51 (61)	127 (35)	222 (16)

**Table 3:** Description of the samples collected and corresponding meteorological conditions.

Sampling Site	Date of collection	Location	Climate	Altitude	Annual precipitation (mm)
Hulunber Grassland	2011-08-08; 2012-05-12; 2012-06-01;2012-07-08; 2012-07-11; 2012-07-302012-08-07; 2013-05-26; 2013-05-28;2013-06-09; 2013-06-27; 2013-07-12;2013-07-25; 2013-08-04; 2013-08-28	49°19′N, 120°03′E;	semi–arid climate	628 m a.s.l.	400
