# Supplementary material for: Evidence for a missing source of efficient ice nuclei
Source: Sci Rep. 2017 Jan 3;7:39673. doi: 10.1038/srep39673 (PMC5206747; doi:10.1038/srep39673)
Supplement: Supplementary Figure S1 [file srep39673-s1.doc]

Appendix A. Supporting information

**Evidence for a missing source of efficient ice nuclei**

Rui Du^1^*, Pengrui Du^1^, Zedong Lu^1^, Weishan Ren^1^, Zongmin Liang^1^, Saisai Qin^1^, Ziming Li^1^, Yaling Wang^1^, and Pingqing Fu^2^

1 College of Resources and Environment, University of Chinese Academy of Science, Beijing 100049, China;

2 State Key of Laboratory of Atmospheric Boundary Physics and Atmospheric Chemistry, Institute of Atmospheric Physics, Chinese Academy of Sciences, Beijing 100029, China

^*^Correspondence should be addressed to [ruidu@ucas.ac.cn](mailto:ruidu@ucas.ac.cn)


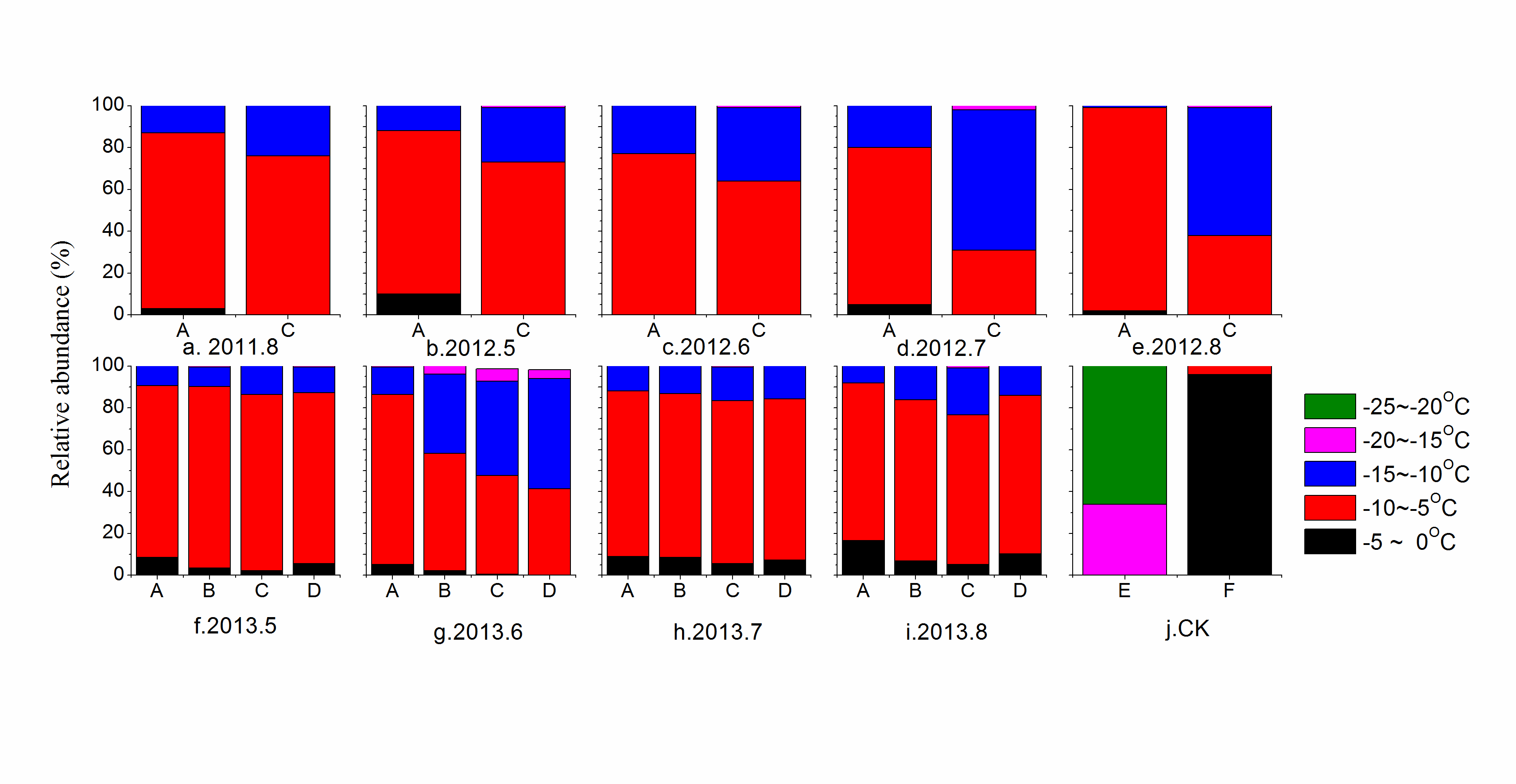


**Supplementary Figure S1 The distribution of frozen droplets under different treatments at temperature interval in 2011, 2012 and 2013.** The capital letter A refers crude rainwater, B refers crude rainwater under heat treatment, C refers filtrate, D refers filtrate under heat treatment, E and F refer ultra-pure water and *P. syringae pv Lachrymans* suspension, respectively
